# Clinical activity of patupilone in patients with pretreated advanced/metastatic colon cancer: results of a phase I dose escalation trial

**DOI:** 10.1038/bjc.2011.438

**Published:** 2011-10-25

**Authors:** B Melichar, E Casado, J Bridgewater, J Bennouna, M Campone, P Vitek, J-P Delord, J Cerman, R Salazar, J Dvorak, C Sguotti, P Urban, K Viraswami-Appanna, E Tan, J Tabernero

**Affiliations:** 1 Charles University Medical School and Teaching Hospital, Sokolská 581, 500 05 Hradec Králové, Czech Republic; 2 Department of Oncology, Palackỳ University Medical School and Teaching Hospital, I.P. Pavlova 6, 775 20 Olomouc, Czech Republic; 3 Medical Oncology Department, Vall d’Hebron University Hospital, Universitat Autònoma de Barcelona, Pl. Vall d’Hebron 119-129, 08035 Barcelona, Spain; 4 University College London Cancer Institute, 72 Huntley Street, London WC1E 6DD, UK; 5 Centre de Recherche en Cancérologie, UMR-INSERM 892, Institut de Cancérologie de l’Ouest/René Gauducheau, Bd. Jacques Monod, 44805 Saint-Herblain, Nantes, France; 6 Institute of Radiation Oncology, Charles University First Medical School, Budinova 2, 180 81 Praha, Czech Republic; 7 Institut Claudius Regaud, 20-24 rue de Pont Saint Pierre, 31052 Toulouse, France; 8 Novartis Pharma AG, Oncology Clinical Development, 4202 Basel, Switzerland; 9 Novartis Pharmaceuticals Corporation, One Health Plaza, East Hanover 07936-1080, NJ, USA

**Keywords:** dose escalation, epothilone B, metastatic colon cancer, microtubule stabiliser, patupilone

## Abstract

**Background:**

New agents that are active in patients with metastatic colorectal cancer are needed. Patupilone (EPO906; epothilone B) is a novel microtubule-stabilising agent.

**Methods:**

Patients with advanced colon cancer who progressed after prior treatment regimens received intravenous patupilone (6.5–10.0 mg m^–2^) once every 3 weeks by a 20-min infusion (20MI), 24-h continuous infusion (CI-1D) or 5-day intermittent 16-h infusion (16HI-5D). Adverse events (AEs), dose-limiting toxicities (DLTs), pharmacokinetics and anti-tumour activity were assessed.

**Results:**

Sixty patients were enrolled. The maximum tolerated dose (MTD) was not reached in the 20MI arm (*n*=31), as no DLTs were observed. Three patients in the CI-1D arm (*n*=26) experienced 1 DLT each at 7.5, 8.0 and 9.0 mg m^–2^, but MTD was not reached. However, the prolonged 16HI-5D arm was terminated at 6.5 mg m^–2^ after two of the three patients developed a DLT. Diarrhoea was the most common AE and DLT, with increased severity at the higher doses (9.0 and 10.0 mg m^–2^). Grade 3 or 4 diarrhoea was observed in 11 (35%) of the patients in the 20MI arm, 4 (15%) of the patients in the CI-1D arm and 2 (67%) of the patients in the 16HI-5D arm. Patupilone activity was observed in the 20MI arm with a disease control rate of 58%, including four confirmed partial responses. The disease control rate in CI-1D arm was 39%.

**Conclusion:**

Patupilone given once every 3 weeks as a 20-min infusion had promising anti-tumour activity and manageable safety profile at doses that demonstrated therapeutic efficacy.

Colorectal cancer (CRC) continues to be one of the most common malignancies worldwide. Despite recent advances in the treatment of CRC, the prognosis of patients with advanced or metastatic disease remains modest. Advances in systemic chemotherapy using fluoropyrimidines, irinotecan and oxaliplatin have increased the median overall survival of patients with metastatic CRC (mCRC) to >20 months (Grothey *et al*, 2004; Meyerhardt and Mayer, 2005), and the development of targeted therapies against epidermal growth factor receptor and vascular endothelial growth factor have translated into further survival improvements (Cunningham *et al*, 2004; Hurwitz *et al*, 2004; Sobrero *et al*, 2008). The objective response rate with the front line use of combination of fluoropyrimidines with irinotecan or oxaliplatin is between 40% and 50%, with median progression-free survival (PFS) duration being around 8 months (Goldberg *et al*, 2004; Tournigand *et al*, 2004). The addition of targeted agents (bevacizumab or cetuximab) to combined chemotherapy results in response rate of up to 60% and median PFS of 10–11 months (Hurwitz *et al*, 2004; Tol *et al*, 2009; Van Cutsem *et al*, 2009). Analysis of the data of randomised clinical trials of first-line therapy further indicates a correlation between response rate, PFS and overall survival (Tang *et al*, 2007). However, most patients with mCRC will ultimately relapse or progress, and the activity of cytoxic or targeted agents administered as monotherapy or in combinations is lower, with the best response rates around 10% in patients treated with a single agent and 20% in patients treated with combinations, and median PFS ranging mostly between 2 and 4 months (Cunningham *et al*, 2004; Tournigand *et al*, 2004; Giantonio *et al*, 2007; Jonker *et al*, 2007). Therefore, new potentially non-cross-resistant agents with novel mechanisms of action are urgently needed.

Patupilone (EPO906; epothilone B) is a novel microtubule-stabilising agent that induces cell-cycle arrest and apoptosis (Bollag *et al*, 1995; Kowalski *et al*, 1997). This mechanism of action is similar to taxanes, but patupilone differs from taxanes in several key aspects. Patupilone is more soluble than taxanes and several times more potent *in vitro*. Most importantly, it is not a substrate for P-glycoprotein and other efflux pumps, and therefore retains activity against cells with a multidrug-resistant phenotype both *in vitro* and *in vivo* (O’Reilly *et al*, 2008). Supporting these preclinical concepts, patupilone has demonstrated activity in taxane-resistant patients (Hussain *et al*, 2009), and the drug is considered to be non-cross-resistant to common cytotoxic agents.

Patupilone has a unique toxicity profile with little or no haematological toxicity and limited neurotoxicity, but significant gastrointestinal toxicity, primarily diarrhoea. Prior clinical trials in patients with advanced CRC using a weekly schedule of patupilone showed modest efficacy, perhaps because the incidence of diarrhoea limited escalation of the dose intensity to a potentially therapeutic level (Poplin *et al*, 2003). We hypothesised that diarrhoea could be a greater problem in patients with previously treated mCRC because of prior chemotherapy or pelvic radiation, bowel resections or nutritional deficits. Consequently, it was proposed that proactive diarrhoea management guidelines that stress the importance of early detection and active symptom control could improve tolerability, increase dose intensity and improve efficacy (Wadler *et al*, 1998; Kornblau *et al*, 2000; Benson *et al*, 2004), as has been shown for irinotecan (Abigerges *et al*, 1994). Moreover, some data indicate that the use of glutamine and other nutritional supplements may help recovery of bowel mucosa (Gibson, 1998; Belluzzi *et al*, 2000; Bjorck *et al*, 2000; Daniele *et al*, 2001; Juntunen *et al*, 2001). It was also hypothesised that prolonged infusion schedules of patupilone could be more effective or better tolerated as has been demonstrated for 5-fluorouracil (5-FU) and irinotecan (Meta-analysis Group in Cancer, 1998; Takimoto *et al*, 2000).

The present phase I study was designed to evaluate the tolerability and maximum tolerated dose (MTD) of a 20-min, 24-h and 5-day infusion of patupilone every 3 weeks, together with prophylactic nutritional supplementation and active diarrhoea management in patients with pretreated mCRC.

## Materials and methods

### Patient eligibility

Patients had histologically confirmed, inoperable locally advanced or metastatic colon cancer progressing after a minimum of one line of therapy with at least one measurable lesion, age ⩾18 years, life expectancy ⩾12 weeks, World Health Organization performance status of 0–1 and no impairment of hepatic or renal function. Initially, the trial was designed to study patupilone as a second-line treatment. Because of significant advances in the second-line therapy of mCRC that resulted in the evolution of standard of care during the conduct of the trial, the protocol was later amended to allow the inclusion of patients with up to four prior lines of chemotherapy. All patients had to have at least one prior line in metastatic setting that included fluoropyrimidines as well as irinotecan and/or oxaliplatin. Prior anti-neoplastic agents other than 5-FU are summarised in Table 1. Main exclusion criteria included brain metastases, ileostomy or colostomy, history of pelvic radiotherapy, grade >1 diarrhoea at baseline and use of prophylactic loperamide. All patients provided written, informed consent and approval was obtained from the ethics committees at the participating institutions and regulatory authorities. The study followed the Declaration of Helsinki and good clinical practice guidelines.

### Study design

Patupilone was administered every 3 weeks either as a 20-min infusion (20MI), 24-h continuous infusion (CI-1D) or 5-day continuous infusion (16-h per day over 5 days; 16HI-5D) with planned dose levels of 6.5, 7.0, 7.5, 8.0, 9.0 and 10.0 mg m^–2^ until disease progression, unacceptable toxicity or withdrawal of consent. A standard 3+3 design was used to determine MTD (Storer, 1989). Initially, three patients were enrolled at each dose level. Dose escalation proceeded in the absence of more than one of six patients with dose-limiting toxicities (DLTs) in the first two cycles of treatment. If two or more patients presented with DLT at a dose level, enrolment of patients to that dose level was discontinued and the immediately preceding dose level was considered the MTD.

### Definition of DLTs

The DLT was defined as any one of the following drug-suspected toxicities (National Cancer Institute Common Toxicity Criteria (NCI-CTC), version 2.0): (a) haematological: grade 2 or 3 neutropenia persisting >2 weeks beyond the scheduled start date of the next cycle; ⩾grade 3 with absolute neutrophils count (ANC) <1000 μl^–1^ and fever ⩾38.5 °C (febrile neutropenia); grade 4 neutropenia with ANC <500 μl^–1^ for ⩾5 days duration; platelet count <20 000 mm^–3^ or need for platelet transfusion; platelet count <75 000 mm^–3^ for >2 weeks beyond the scheduled start date of the next cycle and (b) non-haematological: total bilirubin ⩾2.0 × upper limit of normal (ULN); grade 4 serum glutamic oxaloacetic transaminase/serum glutamate pyruvate transaminase (SGOT/SGPT); grade 3 SGOT/SGPT; any grade 3 nausea or ⩾grade 3 vomiting or diarrhoea persisting for >7 days, despite maximal medical treatment; any other ⩾grade 3 adverse event (AE) (except myalgia and/or arthralgia that responds to symptomatic therapy); creatinine ⩾3.0 × ULN; any ⩾grade 2 neurotoxicity; any death considered related to study drug.

### Diarrhoea management and nutritional supplement

Based on the guidelines for management of chemotherapy-induced diarrhoea (CID) (Wadler *et al*, 1998; Kornblau *et al*, 2000; Benson *et al*, 2004), an algorithm for the diagnosis and treatment of diarrhoea toxicity was established to potentially lessen its severity and duration. In short, patients were proactively contacted to identify the early signs of diarrhoea and provided with dietary recommendations and immediate treatment with loperamide (2 mg every 2 h until the control of diarrhoea was achieved). Unresolved diarrhoea was further treated with opiates and infusion therapy during hospitalisation, as needed.

On the basis of clinical and preclinical data, a nutritional supplement was used that demonstrated a potential beneficial effect on the gut mucosa and bowel function; use of the supplement showed promising results in patients with CID (Gibson, 1998; Belluzzi *et al*, 2000; Bjorck *et al*, 2000; Daniele *et al*, 2001; Juntunen *et al*, 2001). The nutritional supplement was administered once daily in a 250-ml serving that contained omega-3 fatty acids (0.5 g docosahexaenoic acid and 1 g eicosapentaenoic acid), short-chain fructo- (5 g) and galactooligosaccharides (5 g), high-quality egg protein with anti-secretory factor (3 g) and probiotic *Bifidobacterium lactis* (2 g) and glutamine (5–10 g). The administration of nutritional supplement was started 7 days before and continued daily upon initiation of patupilone treatment during the entire course of therapy.

### Safety and response assessments

Routine clinical and laboratory assessments were conducted at baseline, before each treatment and at the end of study visit. Electrocardiograms were performed at baseline and at the end of treatment. AEs were recorded and graded using the NCI-CTC v2.0, and they were assessed by the investigator for any relationship with patupilone treatment.

Objective measurement of tumour mass was assessed in accordance with Response Evaluation Criteria in Solid Tumours v1.0 at baseline and thereafter every 8 weeks. Complete (CR) and partial responses (PR) were to be confirmed at least 4 weeks after the initial declaration of response. Efficacy variables included best overall response and time to progression (TTP).

### Pharmacokinetic assessments

In the 20MI arm, blood samples were collected during cycles 1 and 4 before drug administration, at the end of infusion and 0.5, 1, 2, 4, 8, 24, 168, 336 and 504 h post-infusion start. For the CI-1D arm, samples were collected during cycle 1 before drug administration, at 4, 8 and 24 h (during infusion) and 24.17, 24.33, 24.67, 25, 26, 28, 32, 48, 72, 168, 336 and 504 h post-infusion start. For the 16HI-5D arm, blood samples were collected during cycle 1 before drug administration, at 16, 24, 40, 48, 64, 72, 88, 96 and 112 h (during infusion) and 112.17, 112.33, 112.67, 113, 114, 116, 120, 144, 168, 336 and 504 h post-infusion start.

Patupilone concentrations in blood were analysed by liquid chromatography-tandem mass spectrometry with a detection limit of 0.1 ng ml^–1^ (Forster *et al*, 2007). Pharmacokinetics (PK) of patupilone was determined using a non-compartmental analysis method (Win-Nonlin; Pharsight, Mountain View, CA, USA), and the area under the concentration–time curve (AUC) was calculated by linear trapezoidal method.

## Results

### Patients

A total of 60 patients were enrolled (Table 1); 31 in the 20MI arm, 26 in the CI-1D arm and 3 in the 16HI-5D arm. The mean age for all patients in the study was 59 years and 30 (50%), 17 (28%) and 13 (22%) patients had 1, 2 or ⩾3 prior lines of chemotherapy, respectively. All patients had at least one cycle of patupilone and were eligible for safety and efficacy assessments.

### Treatment administered and safety

The numbers of cycles administered and reasons for discontinuation are detailed in Table 1. DLT was always constituted by persisting grade 3 or 4 diarrhoea. The MTD, as defined by the protocol, was not reached in the 20MI arm because no DLTs were observed. Similarly, the MTD was not reached in the CI-1D arm, although three diarrhoea DLTs (one each at 7.5, 8.0 and 9.0 mg m^–2^ dose levels) were observed (Table 2). The 16HI-5D arm was terminated after two DLTs (diarrhoea) occurred in two of the three patients treated with 6.5 mg m^–2^ patupilone. Three patients died during the study; the cause of death was disease progression (*n*=2; CI-1D) and acute renal failure (*n*=1; 20MI arm).

Gastrointestinal toxicity, mainly diarrhoea, was the most commonly observed AE associated with patupilone administration (Table 3). Diarrhoea (any grade) was noted in 25 (80%) of the patients in the 20MI arm, 19 (73%) of the patients in the CI-1D arm and 3 (100%) of the patients in the 16HI-5D arm. Grade 3 or 4 diarrhoea was observed in 11 (35%) of the patients in the 20MI arm, 4 (15%) of the patients in the CI-1D arm and 2 (67%) of the patients in the 16HI-5D arm. Other common AEs included nausea, vomiting, anorexia, fatigue, abdominal pain and neuropathy. In general, there was an increase in the incidence and severity of AEs as the dose increased. However, with the exception of diarrhoea, few of these events were severe. Of note, little haematological, hepatic or cardiac toxicity was observed. No grade 3/4 events, including diarrhoea, were observed in the 20MI arm until dose 8.0 mg m^–2^. Incidences for two of the most frequent AEs (diarrhoea and neuropathy) as a function of dose are summarised in Table 2. In about half of the patients, AEs, which were most commonly diarrhoea, led to dose adjustment and/or interruption at some point during the treatment. Discontinuation due to AEs occurred in seven (23%), four (15%) and two (67%) patients in the 20MI, CI-1D and 16HI-5D arms, respectively (Table 1).

### PK assessments

**Figure 1:**
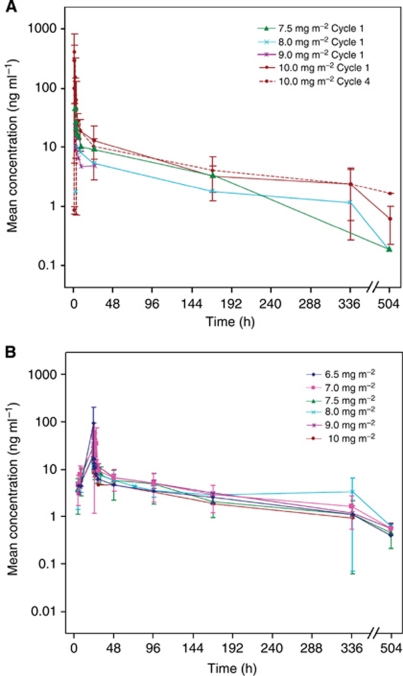
Mean patupilone blood concentration±s.d. (**A**) Cycle 1 and cycle 4 data from 20MI arm. (**B**) Cycle 1 data from CI-1D arm.

Cycle 1 PK samples were available from 10 of 31 patients in the 20MI arm, 22 of 26 patients in the CI-1D arm and all three patients in the 16HI-5D arm. The mean patupilone concentration–time profiles by dose and infusion schedule after the first dose are shown in Figure 1A and B for the 20MI and CI-1D arm, respectively, and PK parameter estimates are summarised in Table 4. Patupilone blood concentration–time profile declined rapidly after infusion, followed by a long terminal half-life of 4–7 days. The steady-state volume of distribution ranged from 430 to 1171 l m^–2^, indicating extensive distribution to tissues. The low blood clearance of patupilone (3–9 l per h per m^2^) was consistent with its long terminal half-life.

Only limited cycle 4 PK data were available (*n*=3; 20MI arm); however, the ratio of AUC (cycle 4/cycle 1) for these few patients was close to 1, suggesting no drug accumulation. The relationship between dose and systemic exposure was inconclusive due to the small PK data set within each arm, large interpatient variability and the small dosing range from 6.5 to 10.0 mg m^–2^. Further, there were no differences in systemic exposure between the 20MI and the CI-1D arm. Similarly, due to the large interpatient variability, the relationship between systemic exposure of patupilone and severe diarrhoea was inconclusive.

### Efficacy assessments

Four confirmed PRs were observed (Table 5), all in the 20MI arm (three PRs at 9.0 mg m^–2^ and one PR at 10.0 mg m^–2^), and an additional unconfirmed PR was reported at 7.5 mg m^–2^ in the 20MI arm. Three out of the four patients with confirmed PR had only one prior line of therapy for metastatic disease (one of these patients had adjuvant chemotherapy), and one patient responded after four prior lines of therapy for metastatic disease. In all responding patients, PR was noted at the first evaluation (after two cycles). Twenty-seven patients (14 (45%), 10 (39%) and 3 (100%) in the 20MI, CI-1D and 16HI-5D arms, respectively) had stable disease (SD) as their best response at doses as low as 6.5 mg m^–2^. The disease control rate (sum of objective response and SD) was 58% (18 of 31 patients) in the 20MI arm and 39% (10 of 26 patients) in the CI-1D arm. In one patient, resection of the residual lesion was performed. This patient was disease free until May 2011 (69 months after the start of patupilone and 62 months after the resection). In May 2011, recurrent liver metastasis were detected, and the patient is currently receiving another systemic therapy. The median TTP was 4.3 months (95% confidence interval: 2.2, 6.2) and 2.0 months (95% confidence interval: 1.9, 3.4) in the 20MI and CI-1D arm, respectively.

## Discussion

The data presented in this manuscript suggest encouraging activity of patupilone monotherapy administered as short-term infusion in patients with mCRC progressing after at least one line of chemotherapy. The confirmed response rate (13%) of patients treated with 20MI patupilone compares favourably with the response rates of currently available drugs used as monotherapy in the second-line setting (1–13%) (Rougier *et al*, 1998; Rothenberg *et al*, 2003; Cunningham *et al*, 1998, 2004). If we consider only patients treated with patupilone doses of 8.0 mg m^–2^ and higher that are thought to represent an active dose range and have been used across the spectrum of indications in phase II or III trial setting, the response rate may be even higher (4 out of 21 patients; 19%). Further, the disease control rate with 20MI, single-agent patupilone was 58%, and long-lasting disease stabilisation was observed at doses as low as 6.5 mg m^–2^, suggesting activity throughout the dose range tested. The median TTP of 4.3 months in the 20MI arm also compares favourably with other second-line agents. It has been demonstrated in patients with mCRC that response rate and PFS are valid surrogates of overall survival (Tang *et al*, 2007). As the survival of mCRC patients has been shown to correlate with the number of active agents available (Grothey *et al*, 2004), the potential of patupilone in this disease should be further explored. Although PRs were observed at higher doses (9.0 and 10.0 mg m^–2^), so too was CID, resulting in potentially more dose adjustments/interruptions. Therefore, lower doses such as 8.0 mg m^–2^ may provide clinical efficacy and be well tolerated, potentially providing a more favourable toxicity/efficacy profile; these could be considered for future studies in this indication.

The promising activity of patupilone observed in the present trial contrasts with the lack of efficacy that was reported in patients with mCRC for another epothilone B analogue, ixabepilone (Eng *et al*, 2004). Compared with patupilone, ixabepilone is more water soluble, but also less cytotoxic (Fumoleau *et al*, 2007). The results of the present trial of patupilone and the phase II trial of ixabepilone demonstrate that both drugs not only may differ in the activity in patients with mCRC, but also have differences in the spectrum of side effects.

In the present trial, the tolerability and MTD of patupilone administered every 3 weeks was assessed using three different infusion schedules. The 5-day 16-h infusion elicited DLTs at the lowest dose tested, 6.5 mg m^–2^, and further exploration was stopped after the first three patients. Higher doses were achieved in the CI-1D arm; however, several DLTs were observed beginning at 7.5 mg m^–2^ and no tumour responses were evident. Although the MTD as defined per protocol was not reached in any of the three arms, comparison of the different schedules indicates that short-term infusion administration may be superior in terms of tolerability, toxicity and anti-tumour activity with no DLTs detected, even at the maximum dose of 10.0 mg m^–2^. Together with the four confirmed responses, this suggests that short-term infusion could be the preferred administration schedule.

The standard of care has significantly changed during the conduct of this trial. The protocol of the present study was designed at the time when the drugs now commonly used for second- and third-line therapy of mCRC, including oxaliplatin, bevacizumab, cetuximab or panitumumab, were either not yet available or the access to these drugs was restricted in some of the countries that participated in the trial, for example, Czech Republic. Subsequently, as these drugs became available throughout the countries participating in the trial, the protocol was amended to reflect the evolution of the standard of care and to allow inclusion of patients with up to four lines of prior systemic therapy for mCRC.

The AEs of patupilone observed in this study were predominantly gastrointestinal and were consistent with the toxicity profile of the drug reported in previous studies (Rubin *et al*, 2005; Forster *et al*, 2007; Hussain *et al*, 2009; Ten Bokkel Huinink *et al*, 2009). In contrast to taxanes and other epothilones, patupilone was not associated with significant haematological toxicity. Although the MTD was not reached in the 20MI and CI-1D arms, the rate of grade 3/4 diarrhoea was increased at the highest dose levels, occurring in 11 of 21 (52%) patients in the 20MI arm treated at doses ⩾8.0 mg m^–2^. This appears higher than the rates previously reported in other indications studied with patupilone. In a similar dose escalation trial of patupilone using the same schedule in patients with relapsed or refractory ovarian, fallopian or primary peritoneal cancer, the highest dose level reached was 11.0 mg m^–2^, and diarrhoea was observed in 87% of the patients, but grade 3 or 4 diarrhoea was only noted in 13% of patients. The rate of grade 3 or 4 diarrhoea in patients treated with a dose of 10.0 mg m^–2^ or higher was 33% (Ten Bokkel Huinink *et al*, 2009). In patients with castration-resistant prostate cancer, the dose of 10 mg m^–2^ had to be decreased to 8 mg m^–2^ because of severe gastrointestinal toxicity observed in four of the six initially enrolled patients. The rate of diarrhoea was 85%, but grade 3 or 4 diarrhoea was observed in 22% of patients (Chi *et al*, 2011). The MTD for weekly administration of patupilone was determined at 2.5 mg m^–2^, and in studies using this schedule, the rate of grade 3 or 4 diarrhoea was reported at 19% and 22% (Rubin *et al*, 2005; Hussain *et al*, 2009). Differences in patient population or chemotherapy schedule may have contributed to the observed differences in the rate of diarrhoea. It is possible that because of prior chemotherapy and bowel resection, patients with mCRC are more susceptible to CID. Despite this, diarrhoea in most cases was manageable and reversible and only a few patients developed dehydration, electrolyte imbalances and acute renal failure as a consequence; although, in one case the renal failure was fatal. Overall, there seemed to be no apparent benefit from using the nutritional supplement in this study; however, there was no control arm and compliance was not optimal.

Improved tolerability of chemotherapeutic schedules is an important goal of drug development. Based on research using 5-FU and irinotecan, prolonged continuous infusion was proposed to have increased anti-tumour effects and less toxicity (Meta-analysis Group in Cancer, 1998; Takimoto *et al*, 2000). Because of these considerations, it was hypothesised that continuous infusion of patupilone could result in improved efficacy and fewer side effects. On the contrary, in the trial presented here, 24-h infusion and 5-day intermittent infusion showed no advantage over short-term infusion in terms of both toxicity and activity. The rather high rates of DLTs at the lowest dose of the 16HI-5D arm suggest efficacy and tolerability profiles for prolonged infusions may be variable and drug specific.

In this trial, since no grade 3 and 4 diarrhoea was observed in the 20MI arm until 8.0 mg m^–2^, further reductions in CID may be achieved through use of lower medication doses. For example, reduction of patupilone from 10.0 to 8.0 mg m^–2^ in prostate cancer patients resulted in dramatic decrease in the incidence of severe diarrhoea while still maintaining encouraging efficacy data (Chi *et al*, 2011). Moreover, since mucosa inflammation may have a role in CID, investigation of anti-inflammatory agents such as steroids for improved tolerability has shown encouraging preclinical results in managing patupilone-induced diarrhoea (McSheehy *et al*, 2008). This strategy has been further explored in clinical trials and indeed emerging data suggest that high-dose prednisone appears to be effective in preventing patupilone-induced diarrhoea and may facilitate treatment with patupilone (Sridhar *et al*, 2010).

Following an intravenous infusion, patupilone was distributed rapidly into tissues, resulting in a large volume of distribution and consistent with the extensive tissue uptake of patupilone observed in animal models (O’Reilly *et al*, 2008). The low blood clearance and long terminal half-life of patupilone were in line with previous phase I studies (Rubin *et al*, 2005; Forster *et al*, 2007; Ten Bokkel Huinink *et al*, 2009). Although only a small number of samples were analysed, there was no evidence of drug accumulation with the 20MI administration of patupilone given every 3 weeks. The large variation in the volume of distribution and clearance of patupilone likely reflect interpatient variability in the tissue and plasma protein binding and biotransformation activities, respectively. Indeed, patupilone is mainly metabolised by carboxylesterases, which have shown large interindividual variability in their activities for various substrates (Hosokawa *et al*, 1995). In this context, the assessment of the relationship between dose and systemic exposure was inconclusive, not only due to a lack of PK data within each arm, but also because of large interpatient variability and a small dosing range from 6.5 to 10.0 mg m^–2^. Accordingly, the relationship between systemic exposure of patupilone and toxicity (e.g. severe diarrhoea) could not be assessed conclusively.

In conclusion, the present data indicate promising activity of patupilone administered as 20-min infusion in patients with previously treated mCRC. The activity of patupilone seems to be comparable to the other second-line therapeutic options in mCRC and deserves further study. CID is a primary toxicity of this therapy. Although the MTD was not reached, reduced doses and/or optimised diarrhoea management protocols may improve dose intensity, warranting further study in this indication.

## Figures and Tables

**Table 1:** Patient demographics and treatment disposition

	**20MI**	**CI-1D**	**16HI-5D**
	***n*=31**	***n*=26**	***n*=3**
	***n* (%)**	***n* (%)**	***n* (%)**
*Gender*			
Male	20 (65)	14 (54)	2 (67)
Female	11 (35)	12 (46)	1 (33)
			
*Age (years)*			
Median	58	60	68
Range	28–81	44–72	60–73
			
*Baseline WHO performance status*			
0	25 (81)	12 (46)	2 (67)
1	6 (19)	14 (54)	1 (33)
			
*Number of prior chemotherapy regimens (including adjuvant)*			
1	16 (52)	13 (50)	1 (33)
2	6 (19)	9 (35)	2 (67)
3	4 (13)	1 (4)	–
4	4 (13)	2 (8)	–
5	1 (3)	1 (4)	–
			
*Prior therapy types (including adjuvant)*			
Irinotecan	23 (74)	18 (69)	3 (100)
Oxaliplatin	19 (61)	13 (50)	0 (0)
Capecitabine	4 (13)	3 (12)	0 (0)
Raltitrexed	2 (6)	0 (0)	0 (0)
Cetuximab	8 (26)	6 (23)	0 (0)
Bevacizumab	1 (3)	3 (11)	0 (0)
Investigational (EKG598)	3 (10)	0 (0)	0 (0)
			
*Number of patupilone cycles received*			
Median	4.0	3.0	2.0
Range	1–21	1–9	2–6
Completed ⩾6 cycles	13 (42)	6 (23)	1 (33)
			
*Reasons for discontinuation*			
Adverse event(s)	7 (23)	4 (15)	2 (67)
Disease progression	22 (71)	20 (77)	1 (33)
Death			
Study indication	—	2 (8)	—
Other causes	1 (3)^a^	—	—
Satisfactory response	1 (3)^b^	—	—

Abbreviations: CI-1D=24-h continuous infusion; WHO=World Health Organization; 16HI-5D=5-day intermittent 16-h infusion; 20MI=20-min infusion.

a Acute renal failure.

b Patient discontinued after nine cycles due to satisfactory response.

**Table 2:** Most frequent drug-related grade 3/4 adverse events and cycle 1 and 2 dose-limiting toxicities

	**20MI**				**CI-1D**				**16HI-5D**			
**Dose (mg m^–2^)**	**Pts in cohort**	**Diarrhoea**	**Neuropathy**	**DLTs**	**Pts in cohort**	**Diarrhoea**	**Neuropathy**	**DLTs**	**Pts in cohort**	**Diarrhoea**	**Neuropathy**	**DLTs**
6.5	4	—	—	—	4	—	—	—	3	2	—	2^a^
7.0	3	—	—	—	4	1	—	—	—	NA	NA	NA
7.5	3	—	—	—	4	1	—	1^a^	—	NA	NA	NA
8.0	3	1	—	—	7	1	—	1^a^	—	NA	NA	NA
9.0	6	4	—	—	7	1	1	1^a^	—	NA	NA	NA
10.0	12	6	3	—	—b	NA	NA	NA	—	NA	NA	NA
Total	31	11	3	—	26	4	1	3^a^	3	2	—	2^a^

Abbreviations: CI-1D=24-h continuous infusion; DLTs=dose-limiting toxicities; MTD=maximum tolerated dose; NA=not applicable; 16HI-5D=5-day intermittent infusion.

a Type of DLT, diarrhoea.

b Although MTD not reached, 10.0 mg m^–2^ dose was cancelled due to increased toxicity and lower efficacy as compared with the 20MI arm.

**Table 3:** Most common adverse events attributed to patupilone (at least 10% cumulative incidence or at least 1 grade 3/4 event)

	**20MI (*n*=31)**		**CI-1D (*n*=26)**		**16HI-5D (*n*=3)**	
	***n* (%)**		***n* (%)**		***n* (%)**	
**Adverse event**	**Grade 1/2**	**Grade 3/4**	**Grade 1/2**	**Grade 3/4**	**Grade 1/2**	**Grade 3/4**
*General*						
Anorexia	5 (16)	—	—	—	—	—
Asthenia/fatigue	3 (10)	1 (3)	4 (15)	—	—	—
Dehydration	2 (6)	1 (3)	1 (4)	1 (4)	1 (33)	—
Pain in extremity	—	1 (3)	—	—	—	—
						
*Gastrointestinal*						
Diarrhoea	14 (45)	11 (35)	15 (58)	4 (15)	1 (33)	2 (67)
Nausea	6 (19)	1 (3)	1 (4)	—	—	—
Vomiting	7 (23)	1 (3)	1 (4)	—	—	—
Abdominal pain	4 (13)	—	3 (12)	—	—	—
Flatulence	5 (16)	—	—	—	—	—
						
*Neurological*						
Neuropathy^a^	6 (19)	3 (10)	3 (12)	1 (4)	—	—
						
*Liver*						
Increased transaminases^b^	—	—	—	1 (4)	—	—

Abbreviations: CI-1D=24-h continuous infusion; 16HI-5D=5-day continuous infusion.

a Peripheral neuropathy (sensory and motor).

b Patient had liver metastasis.

**Table 4:** Summary of pharmacokinetic parameters for patupilone

**Regimen; dose group**	***n***	***C*_max_ (ng ml^–1^)**	***C*_min_ (ng ml^–1^)**	***C*_max_/*C*_min_**	**AUC_(0–∞)_ (ng h ml^–1^)**	***T*_1/2_ (h)**	**CL (l per h per m^2^)**	***V*_ss_ (l m^–2^)**	***R* a**
*20MI*									
7.5 mg m^–2^	1	45.4	0.2	238.9	1777.3	87.0	4.2	430.3	NA^b^
8.0 mg m^–2^	1	9.9	0.2	53.6	1007.1	105.2	8.0	1029.2	NA
9.0 mg m^–2^	1	22.7	NA^b^	NA	NA	NA	NA	NA	NA
10.0 mg m^–2^	7	299.4±243.7^c^	0.6±0.4 (*n*=3)	648.9±342.3	3096.6±2227.4	113.8±46.4	4.5±2.3	547.8±372.9	1.2±0.4^d^
									
*CI-1D*									
6.5 mg m^–2^	2	93.4	0.4 (*n*=1)	435.4	2478.4	132.1	3.2	266.3	NA
7.0 mg m^–2^	3	60.5±19.2	0.5 (*n*=1)	73.6	2120.0±670.9	151.6±27.6	4.7±1.6	641.6±361.3	NA
7.5 mg m^–2^	4	30.5±21.7	0.5 (*n*=2)	65.2	1452.1±780.9	128.7±27.6	7.6±3.9	983.0±483.3	NA
8.0 mg m^–2^	6	26.4±8.2	0.6 (*n*=2)	36.6	1327.2±520.6	123.2±78.8	8.8±5.5	1044.1±423.5	NA
9.0 mg m^–2^	7	31.3±14.6	0.6±0.2 (*n*=4)	69.5±38.7	1622.1±623.8	118.4±24.1	7.4±4.5	920.9±354.4	NA
									
*16HI-5D*									
6.5 mg m^–2^	3	8.7±2.6	0.4 (*n*=2)	27.9	952.6±192.3	117.9±17.8	8.3±1.7	1170.7±303.3	NA

Abbreviations: AUC_(0–∞)_=area under the concentration–time curve from zero to infinity; CI-1D=24-h continuous infusion; CL=blood clearance of patupilone; *C*_max_=peak of the blood concentration of patupilone; *C*_min_=trough concentration of patupilone at ∼504 h (some patients may not have the trough concentration); *C*_max_/*C*_min_=ratio of *C*_max_ to *C*_min_; *T*_1/2_=terminal half-life of patupilone; *V*_ss_=steady-state volume of distribution; 16HI-5D=5-day 16-h infusion.

a *R*, drug accumulation (calculated as AUC_(0–tau)_ fourth dose/AUC_(0–tau)_ first dose).

b No data or not enough data for determining the pharmacokinetic parameters.

c Arithmetic mean±s.d.

d Only three of the seven patients (20MI 10.0 mg m^–2^) have PK data in cycle 4 (or the fourth dose).

∼95% of patupilone is bound to plasma protein.

**Table 5:** Efficacy data

	**20MI**	**CI-1D**
	***n*=31**	***n*=26**
	***n* (%)**	***n* (%)**
*Best overall response*		
Complete response (CR)	0	0
Partial response (PR)	4 (13)	0
Stable disease (SD)	14 (45)	10 (39)
Progressive disease (PD)	11 (35)	14 (54)
Unknown	2 (6)	2 (8)
		
Median TTP (months, 95% confidence interval)	4.3 (2.2, 6.2)	2.0 (1.9, 3.4)

Abbreviations: CI-1D=24-h continuous infusion; TTP=time to progression; 20MI=20-min infusion.
